# An Update on Dermatomyositis and Related Inflammatory Myopathies: Cutaneous Clues, Skeletal Muscle Involvement, and Advances in Pathogenesis and Treatment

**DOI:** 10.3390/muscles4040058

**Published:** 2025-12-01

**Authors:** Andres Parga, Dhruv Ratra, Dana Luu

**Affiliations:** Hospital Corporation of America, Florida Healthcare, Graduate Medical Education, Morsani College of Medicine, University of South Florida, 11375 Cortez Blvd, Brooksville, FL 34613, USA

**Keywords:** dermatomyositis, inflammatory myopathy, cutaneous manifestations, myositis-specific autoantibodies, muscle weakness, immunopathogenesis, targeted therapy, malignancy screening

## Abstract

Dermatomyositis (DM) is a prototypic idiopathic inflammatory myopathy in which characteristic skin disease frequently precedes or parallels muscle involvement and signals risks such as interstitial lung disease (ILD) and malignancy. This literature review integrates recent advances across dermatology, neuromuscular medicine, and immunology to refine diagnosis and management. We surveyed the literature from 2000 to 2025, prioritizing randomized trials, large cohorts, and translational studies that spanned classic and juvenile DM, amyopathic/hypomyopathic variants, and overlap phenotypes. Key insights include the diagnostic weight of pathognomonic cutaneous lesions with nailfold microangiopathy; the utility of myositis-specific autoantibodies for endotyping and risk (e.g., anti-TIF1-γ/anti-NXP2 and cancer, anti-MDA5 and rapidly progressive ILD); and the value of myxovirus-resistance protein A (MxA) immunohistochemistry and muscle MRI patterning (including distinctions from immune-mediated necrotizing myopathy) when enzymes are normal, or biopsies are treatment-modified. Management is anchored in early steroid-sparing immunosuppression tailored to phenotype, with evidence for IVIG in active DM and growing support for JAK inhibition, particularly in interferon-high or anti-MDA5 ILD, alongside selective use of calcineurin inhibitors and rituximab, with plasma exchange considered for refractory, rapidly progressive ILD. We highlight risk-stratified malignancy screening (IMACS 2023) and complications, including calcinosis, lipodystrophy, and chronic cutaneous damage. Skin-led recognition coupled with antibody-guided, phenotype-directed therapy and interdisciplinary care offers a pragmatic precision framework to improve outcomes and reduce long-term disability.

## 1. Introduction

Dermatomyositis is a prototypic idiopathic inflammatory myopathy characterized by distinctive cutaneous manifestations and variable skeletal muscle inflammation and weakness. Within the contemporary idiopathic inflammatory myopathy (IIM) spectrum, dermatomyositis, anti-synthetase syndrome, immune-mediated necrotizing myopathy, inclusion body myositis, and the now-narrowed entity of polymyositis, classification has shifted from clinicopathologic labels toward serology- and phenotype-informed groupings, formalized by the 2017 EULAR/ACR criteria [1,2,3]. Cutaneous findings often provide the earliest and most specific clues to muscle disease. Pathognomonic or highly characteristic lesions, such as Gottron papules/sign, heliotrope rash, shawl/V-sign poikiloderma, periungual capillary changes, and pruritic, photo-distributed plaques, can precede objective myopathy by months, and in clinically amyopathic dermatomyositis define a skin-predominant phenotype with important systemic risks (for example, interstitial lung disease and malignancy) despite minimal or absent muscle weakness. Recognizing these skin patterns enables earlier diagnosis, risk stratification, and targeted testing [4,5]. At a mechanistic level, dermatomyositis is strongly linked to type I interferon-driven microangiopathy-driven vascular injury, with disease activity tracking interferon-stimulated gene signatures and biomarkers (i.e., Siglec-1). Antibody subsets are mapped to distinct clinical trajectories: anti-TIF1-γ and anti-NXP2 are associated with malignancy, while anti-MDA5 is associated with vasculopathic skin disease and rapidly progressive interstitial lung disease, particularly in amyopathic or hypomyopathic presentations. These skin–muscle–autoantibody relationships underpin a precision approach to diagnosis and therapy [6,7,8,9]. Epidemiologically, dermatomyositis is rare. Recent population-based data estimate an incidence of around ~1.1 per 100,000 person-years and a prevalence near ~13 per 100,000 in adults, with female predominance and a bimodal age distribution (juvenile and mid-to-late adulthood). U.S. administrative cohorts report ranges between ~0.1 and 1.8 per 100,000 person-years, suggesting geographic and methodological variability. The burden of morbidity stems not only from muscle weakness but also from interstitial lung disease (ILD), calcinosis (notably in juvenile disease), and cancer-associated risks that peak within several years of disease onset and are highest in anti-TIF1-γ positive patients. These features make dermatomyositis highly relevant to both dermatology and neuromuscular medicine and advocate for coordinated, cross-specialty care [10,11,12,13].

## 2. Overview

### 2.1. Classification and Subtypes

Modern classification of IIMs integrates clinical phenotype, histopathology, and myositis-specific autoantibodies (MSAs) (Table 1). The 2017 EULAR/ACR criteria formalized data-driven subgrouping for adult and juvenile IIM and remain the anchor framework, now increasingly refined by MSA-defined endotypes [1,3,14].

Dermatomyositis (DM): Classic DM is characterized by pathognomonic cutaneous lesions (e.g., Gottron papules/sign, heliotrope) with symmetric, proximal > distal muscle weakness and perifascicular pathology on biopsy. Common MSAs include anti-Mi-2, anti-TIF1-γ, anti-NXP2, anti-SAE, and anti-MDA5, each linked to distinct risks (e.g., malignancy with TIF1-γ/NXP2; RP-ILD with MDA5) [9,15,16,17]. Amyopathic DM (ADM) denotes classic DM skin disease for ≥6 months without objective muscle weakness. Hypomyopathic DM (HDM) adds subclinical muscle involvement by enzymes/EMG/MRI but with minimal or no weakness. These entities carry meaningful systemic risk (e.g., ILD, malignancy) despite absent/overt muscle weakness, underscoring the skin–muscle continuum [18,19].

Clinically amyopathic dermatomyositis (CADM): Often MDA5-positive, CADM presents with hallmark DM rashes with little to no weakness; it is disproportionately associated with vasculopathic skin disease and rapidly progressive ILD, demanding early recognition and aggressive evaluation [9].

Juvenile dermatomyositis (JDM): JDM shares core features with adult DM but exhibits unique complications such as calcinosis cutis (especially in prolonged/undertreated disease) and lipodystrophy. Antibody-phenotype patterns broadly mirror those in adults, with NXP2 linked to calcinosis and MDA5 to ILD in pediatric cohorts [14].

Overlap syndromes: Overlap myositis denotes IIM coexisting with another connective tissue disease. A prominent example is scleromyositis (systemic sclerosis–myositis overlap), frequently associated with anti-PM/Scl or anti-Ku and characterized by distinct clinical and histopathological signatures; recognition is increasing, and some authors argue it is a discrete entity within both spectra [20,21].

Comparison with other major IIM entities: Anti-synthetase syndrome (ASyS): Defined by antibodies to aminoacyl-tRNA synthetases (most commonly anti-Jo-1) with a triad of myositis, interstitial lung disease, and inflammatory arthritis; mechanic’s hands and Raynaud phenomenon are frequent. ILD often dominates prognosis [22,23,24]. Immune-mediated necrotizing myopathy (IMNM): Presents with severe proximal weakness, markedly elevated CK, and myofiber necrosis with scant inflammation; two-thirds of patients harbor anti-SRP or anti-HMGCR antibodies (the latter often statin-exposed) [25,26,27]. Polymyositis (PM): Now considered rare after reclassification of many legacy cases to IMNM, ASyS, inclusion body myositis (IBM), or overlap myositis; PM should be a diagnosis of exclusion [28]. Among the rarer inflammatory myopathies, anti-mitochondrial antibody (AMA)–positive myositis has gained attention as a distinct clinicopathologic subtype. It often presents as chronic skeletal muscle involvement and characteristic cardiac manifestations, including myocarditis, arrhythmia, and conduction abnormalities, underscoring the need for multidisciplinary surveillance [14,20,21]. Recognition of this entity expands the IIM spectrum beyond traditional serotypes and highlights the growing importance of integrating autoantibody profiling into patient evaluation.

**Table 1:** Practical comparison (phenotype, antibodies, and key risks).

Entity	Typical Skin Findings	Muscle Pattern/Pathology	Key MSAs	Salient Risks/Associations
Classic DM	Gottron papules/sign, heliotrope, photo-poikiloderma, periungual changes.	Symmetric proximal weakness; perifascicular atrophy.	Mi-2, TIF1-γ, NXP2, SAE	Malignancy (TIF1-γ/NXP2), calcinosis (NXP2), often good steroid responsiveness (Mi-2) [16,17].
ADM/HDM (incl. CADM)	DM rashes with little/no weakness.	Normal strength (ADM) ± subclinical involvement (HDM).	MDA5 common in CADM	RP-ILD, vasculopathic skin ulcers/palmar papules (MDA5) [9,18].
JDM	As above, more edema and nodules.	Proximal weakness; chronic course.	NXP2, MDA5, Mi-2	Calcinosis, lipodystrophy; ILD subset (MDA5) [14].
ASyS	“Mechanic’s hands,” fissuring; sometimes mild DM-like rash.	Myositis ± mild; ILD often predominant.	Anti–Jo-1 (most), PL-7, PL-12, OJ, EJ	Progressive ILD, arthritis, Raynaud; response varies by antibody [22,23,24].
IMNM	Usually nonspecific.	Severe necrotizing myopathy, very high CK.	Anti-SRP, anti-HMGCR	Statin association (HMGCR), chronic weakness if delayed treatment [25,27].
Scleromyositis (overlap)	Scleroderma skin changes; nailfold capillary drop-out.	Variable myopathy with SSc features.	PM/Scl, Ku	Cardiorespiratory involvement; distinct biopsy patterns; management per overlap [21].
Anti-mitochonrial antibody myositis	Often subtle or absent; may show nonspecific erythema or DM-like rash.	Chronic myopathy with necrotizing or granulomatous features; frequent cardiac involvement (myocarditis, arrhythmia, conduction block); possible mild CK elevation.	AMA (mainly M2)	Cardiac dysfunction and arrhythmia; overlap with autoimmune cholangitis; may respond to immunosuppression ± cardiac management [14,20,21].
Polymyositis (exclusion)	Nonspecific	Endomysial CD8+ T-cell–mediated myofiber invasion (rare).	—	Reassess for IMNM/ASyS/IBM/overlap before labeling PM [28].

### 2.2. Cutaneous Manifestations

Pathognomonic lesions: The two pathognomonic findings are Gottron’s papules/sign, violaceous to pink papules/plaques or macular erythema over extensor joints (metacarpophalangeal, interphalangeal, elbows, knees), and the heliotrope rash, which is a violaceous periorbital erythema often associated with eyelid edema (Table 2). These lesions are highly specific for dermatomyositis (DM) and may precede weakness by months [4].

Highly characteristic (non-pathognomonic) patterns: Photo-accentuated poikiloderma and erythema may localize to the shawl sign (posterior neck/shoulders/upper back), V-sign (anterior chest/neck), and the holster sign (violaceous erythema or poikiloderma over the lateral thighs) (Table 3). The holster sign is less photosensitive than shawl/V and is easily overlooked [29,30].

Periungual and nailfold changes: Microangiopathy produces dilated and tortuous capillary loops, capillary “drop-out,” periungual erythema, and ragged cuticles; these changes correlate with disease activity and can be followed noninvasively with dermoscopy or nailfold videocapillaroscopy. Recent cohorts show abnormal nailfold patterns in most patients at presentation, and emerging work suggests associations with cytokine profiles and interstitial lung disease risk [31,32,33].

Pruritus and poikiloderma: Pruritus is common and can be disabling in DM; mechanistic studies implicate the IL-31 axis among pruritogens. Poikiloderma-reticulated hypo/hyperpigmentation with telangiectasias and epidermal atrophy often accompanies photo-distributed diseases (e.g., shawl/V) and contributes to chronic cutaneous damage [34].

Skin histopathology: Typical biopsies show vacuolar interface dermatitis with epidermal atrophy, basement-membrane thickening, increased dermal mucin, and a superficial perivascular lymphocytic infiltrate; the pattern may resemble lupus. Complement (C5b-9) deposition has been demonstrated on dermal vessels/dermo-epidermal junction in skin and on endomysial capillaries in muscle, reflecting microvascular injury in DM [22,35,36,37].

### 2.3. Skeletal Muscle Involvement

Dermatomyositis (DM) classically produces symmetric, proximal-predominant weakness of the shoulder/hip girdles and neck flexors; bulbar and (less often) respiratory muscle involvement can complicate severe disease (Table 4). Importantly, strength and CK may be near-normal in hypomyopathic and anti-MDA5 phenotypes; recent case-based syntheses show anti-MDA5 DM may present without rash or weakness yet rapidly develop ILD, underscoring the need for imaging and tissue confirmation when clinical suspicion persists [38].

Muscle biopsy: The stereotyped lesion is a perifascicular microangiopathy, perifascicular fiber atrophy with perivascular/perimysial lymphocytic inflammation, capillary rarefaction, and membrane–attack complex (C5b-9) deposition on endomysial microvessels (sometimes sarcolemma), supporting vascular injury as a central mechanism [22]. Comparative pathology reviews reaffirm these features as discriminative for DM within the IIM spectrum [39]. Overexpression of MHC-I on the sarcolemma and the presence of scattered necrotic and regenerating fibers are general histologic features shared across IIMs and should be noted during biopsy interpretation.

Interferon signature and MxA immunohistochemistry: Routine use of myxovirus-resistance protein A (MxA) staining now helps separate DM from other IIMs, including treatment-modified biopsies. In a 138-specimen series, sarcoplasmic MxA showed 46% sensitivity and 94% specificity for DM (perifascicular pattern in all positives), with prior cohorts reporting ~57–77% sensitivity and 98–100% specificity; recent guidance therefore recommends adding MxA to standard biopsy panels while interpreting results alongside morphology to avoid pitfalls [40,41].

MRI correlations: Muscle MRI sensitively detects subclinical activity and guides biopsy: active disease shows bilateral, symmetric T2/STIR hyperintensity (edema) in proximal girdles and often fascia; T1 hyperintensity/atrophy reflects chronic damage. Short tau inversion recovery (STIR) hyperintensity is not specific to dermatomyositis. It may also be seen in active stages of muscular dystrophies or denervation injury, typically with a distinct distribution pattern. Contemporary pattern-recognition work demonstrates that DM and immune-mediated necrotizing myopathy (IMNM) involve different thigh muscle sets, in a 2025 cohort (DM *n* = 11; IMNM *n* = 14), DM preferentially affected vastus lateralis/intermedius/medialis, rectus femoris, semitendinosus, and biceps femoris-short head (plus gracilis/sartorius), whereas IMNM favored semimembranosus, biceps femoris-long head, and adductors; 10/11 muscles differed (*p* < 0.01), improving noninvasive phenotyping and biopsy site selection [42,43].

EMG correlations: Needle EMG typically shows a myopathic pattern (short-duration, low-amplitude motor unit potentials with early recruitment), plus fibrillation/positive sharp waves during active disease. Because EMG sensitivity is imperfect and may be normal early, it is best used with MRI for localization rather than as a stand-alone rule-out test [44].

Bulbar involvement (dysphagia): Swallowing muscles are frequently affected across IIMs. In a 2024 single-center cohort (*n* = 108 IIM), 16.7% developed dysphagia and 72.2% achieved complete oral intake within one year, yet survival was significantly worse when malignancy co-occurred, and the data reinforces the need for early oncologic evaluation and multidisciplinary swallow rehabilitation in at-risk DM subgroups [45].

Juvenile versus adult disease: Juvenile DM mirrors the proximal, symmetric weakness of adults but shows more prominent vasculopathy/fascial and subcutaneous edema on MRI and higher risks of calcinosis and lipodystrophy; malignancy association is rare. Whole-body MRI (WB-MRI) in contemporary pediatric cohorts detects myositis lesions and correlates with CK elevation, supporting its role as a problem-solving tool in JDM/juvenile IIM. Emerging pediatric data also highlights type-I-IFN-linked biomarkers (e.g., CXCL10, galectin-9) that often outperform CK for activity assessment [46,47].

Clinical takeaway: DM muscle involvement is best characterized by an interferon-driven perifascicular microangiopathy on biopsy, STIR-bright muscle/fascial edema on MRI, and a proximal, symmetric weakness pattern clinically. Integrating serology, MxA IHC, MRI patterning, and targeted EMG yields the most accurate diagnosis. This is especially true in phenotypes such as anti-MDA5 or hypomyopathic disease, preventing missed or delayed recognition of serious extramuscular complications [38,40,43].

**Table 4:** Skeletal muscle involvement in dermatomyositis. Summary of key studies linking clinical examination, biopsy with MxA immunohistochemistry, muscle MRI, and EMG for diagnosis, phenotyping, biopsy targeting, and prognosis. Rows report population/design, principal muscle or biomarker finding, and clinical implications. Abbreviations: DM = dermatomyositis; IMNM = immune-mediated necrotizing myopathy; IIM = idiopathic inflammatory myopathy; MxA = myxovirus-resistance protein A; WB-MRI = whole-body MRI; JDM = juvenile dermatomyositis; JIIM = juvenile idiopathic inflammatory myopathy; ILD = interstitial lung disease; RP-ILD = rapidly progressive ILD; NSIP = nonspecific interstitial pneumonia.

Study (Year)	Population/Design	Key Muscle Finding (Method)	Clinical Implication
Wilks et al. (2025) [43]	DM vs. IMNM, MRI cohort	Distinct thigh muscle involvement patterns separate DM from IMNM (anterior vs. posterior/adductor predominance).	Improves phenotyping and MRI-guided biopsy site selection.
Uruha et al. (2016) [48]	IIM biopsy series	Sarcoplasmic MxA IHC accurately identifies DM.	Add MxA to routine biopsy panels; supports interferon-driven DM endotype.
Xing et al. (2024) [49] Waisayarat et al. (2023) [40]	Multicenter pathology cohorts	High DM specificity of MxA across myositides; notes interpretation pitfalls.	Reinforces diagnostic utility; informs labs on staining protocols.
Ohmura et al. (2024) [45] Leclair et al. (2024) [50]	IIM dysphagia cohorts	~17% dysphagia; outcomes poorer when cancer present.	Screen for malignancy; prioritize multidisciplinary swallow care.
Xie et al. (2024) [51] Bombardi et al. (2024) [52]	293-patient antibody cohort + NXP2 case	NXP2 associates with myalgia/weakness/dysphagia; severe, refractory bulbar disease reported.	Antibody-informed risk counseling; early swallow evaluation in NXP2+.
Ozga et al. (2024) [46]	Pediatric WB-MRI series	WB-MRI detects muscle and fascial edema; correlates with labs.	Use WB-MRI as a sensitive tool in JDM/JIIM workups.
Xiao et al. (2024) [38] Nombel et al. (2021) [53]	Anti-MDA5 case + review	MDA5 DM can lack rash/weakness, present with RP-ILD.	Do lung imaging and serology even when CK/strength are normal.
Zhao et al. (2025) [54]	40 anti-MDA5 DM-ILD pts	NSIP pattern and inflammatory markers predict mortality; combined immunosuppression improves survival.	Supports early aggressive therapy and prognostic stratification.

### 2.4. Immunopathogenesis and Malignancy Association

Dermatomyositis (DM) is a prototypic Type I interferon (IFN)-driven disease. Bulk and single-cell datasets demonstrate robust IFN-stimulated gene (ISG) signatures across muscle, skin, endothelium, and blood, with higher ISG scores tracking organ activity and autoantibody subsets (particularly for anti-MDA5). Mechanistic and translational work further implicates IFN-β as a dominant circulating/skin signal, and clinical studies show that JAK-STAT inhibition can blunt IFN-induced tissue damage [8,55,56,57]. For clarity, several key terms used throughout this review are briefly defined. Perifascicular atrophy refers to the selective thinning of muscle fibers at the outer edges of fascicles, which is a hallmark feature of dermatomyositis. The type I interferon signature describes the upregulation of interferon-stimulated genes that drive inflammation and vasculopathy, while microangiopathy denotes the small-vessel injury and complement-mediated capillary drop-out characteristic of the disease process [6,35].

Myositis-specific autoantibodies (MSAs) and clinicobiologic endotypes [58] are as follows:

- Anti-Mi-2: classic photo-accentuated rash with generally favorable steroid responsiveness; cancer risk is not clearly increased (classified as intermediate/standard by IMACS).
- Anti-TIF1-γ: strongest and most reproducible association with malignancy in adult IIM—IMACS meta-analysis estimates RR ≈ 4.7 vs. other IIM; risk rises after age 40 and may be amplified by additional “high-risk” clinical features (e.g., dysphagia, ulceration).
- Anti-NXP2: links to calcinosis (children) and, in adults, a possible increase in cancer risk, with evidence for this being mixed; the IMACS panel still categorizes NXP2 positivity as high-risk for screening purposes.
- Anti-MDA5: vasculopathic phenotype (ulcerations, palmar papules) with rapidly progressive ILD, often amyopathic/hypomyopathic; malignancy risk is not elevated.

DM muscle shows a perifascicular microangiopathy characterized by capillary loss and C5b-9 (membrane–attack complex) deposition on endomysial microvessels, with findings reproduced across adult and juvenile cohorts. Later analyses suggest MAC assembly may be a downstream marker of microvascular injury rather than the initiating event, aligning with IFN-endothelial biology [35,59]. Parallel IFN signatures in skin and muscle (and nailfold microangiopathy mirroring systemic vasculopathy) explain why cutaneous activity frequently anticipates or parallels myopathy. This cross-tissue program underpins the diagnostic utility of MxA immunohistochemistry in both muscle and skin and supports a shared endothelial–immune axis driving DM pathology [8,57]. Adult-onset DM carries the largest cancer risk among IIMs, particularly within ±3 years of myositis onset. IMACS risk stratification designates DM subtype, anti-TIF1-γ positivity, anti-NXP2 positivity, age >40 years, persistent high disease activity, dysphagia, and skin necrosis/ulceration as high-risk factors, with anti-MDA5, anti-Mi-2, anti-SAE, male sex, and certain IIM subtypes designated as intermediate-risk factors [58]. Across population and meta-analytic data, the tumors most repeatedly linked to DM include ovarian, lung, gastrointestinal (especially colorectal), and breast cancers; melanoma, non-Hodgkin lymphoma, and nasopharyngeal carcinoma contribute in specific regions/ancestries [60,61].

Screening recommendations (adult IIM, IMACS 2023) [58]:

- Risk-stratify at diagnosis using subtype, MSA, and clinical features (high, moderate, standard).
- All patients: perform basic screening plus country-specific age/sex-appropriate cancer screening. (Basic panel includes history/physical, CBC, LFTs, inflammatory markers, SPEP/light chains, urinalysis.)
- High-risk patients: add enhanced screening (commonly CT chest/abdomen/pelvis ± tumor-directed tests). If unrevealing, consider 18F-FDG PET/CT as a single-step screen, especially in anti-TIF1-γ DM > 40 years with ≥1 additional high-risk feature, and consider upper/lower endoscopy where GI risk is prominent.
- Geography-specific: in East/Southeast Asian populations, consider nasoendoscopy due to higher nasopharyngeal carcinoma prevalence.
- Time window: focus highest screening intensity within the first 3 years after symptom onset (when most cancers cluster), with continued vigilance thereafter.

DM integrates a type I IFN-centered immune program, microvascular injury, and MSA-defined endotypes that are mapped to organ involvement and cancer risk. In practice, pairing serology with MxA-supported histology and risk-stratified cancer screening (IMACS) enables earlier tumor detection and more personalized monitoring.

### 2.5. Diagnostic Approach

#### 2.5.1. Integrated Dermatology–Neuromuscular Evaluation

Start with a focused skin exam (pathognomonic lesions, photo-accentuated patterns, nailfolds) and a neuromuscular exam targeting symmetric proximal weakness, dysphagia, and respiratory symptoms. Contemporary guidance emphasizes correlating clinical phenotype + serology + histology rather than relying on any single element, building on the EULAR/ACR framework for IIM classification. Nailfold videocapillaroscopy (NVC) is a useful, noninvasive adjunct: capillary density and morphology vary with activity in adult DM cases and several groups now argue NVC features should be incorporated into diagnostic criteria [41,62,63].

#### 2.5.2. Laboratory Workup

Order a myositis lab panel with CK, aldolase, LDH, AST/ALT, and MSA/MAA serologies. Remember that CK can be normal in dermatomyositis, especially hypomyopathic and anti-MDA5 phenotypes, and isolated aldolase elevation can be the only biochemical clue to active myopathy; recent series reinforce aldolase testing to increase diagnostic sensitivity when CK is normal. Inflammatory markers (ESR/CRP) are variably elevated and nonspecific [64].

#### 2.5.3. Imaging

Muscle MRI is the preferred imaging test for suspected IIM because it detects subclinical activity, distinguishes active edema (T2/STIR hyperintensity) from chronic damage (T1 fatty replacement/atrophy), and guides biopsy to the most inflamed muscle. Recent pattern-recognition work showed DM and immune-mediated necrotizing myopathy (IMNM) affect distinct thigh muscle groups; DM favors anterior compartment (vasti/rectus) and semitendinosus/short-head biceps (plus gracilis/sartorius), whereas IMNM favors semimembranosus, long-head biceps, and adductors, supporting MRI for both phenotyping and site selection [43,65].

#### 2.5.4. Electrodiagnostics

EMG typically demonstrates a myopathic pattern (short-duration, low-amplitude MUAPs with early recruitment; fibrillations/positive sharp waves in active disease) but may appear to be normal early on; use EMG with MRI to localize an optimal biopsy target rather than to exclude disease [41].

#### 2.5.5. Skin and Muscle Biopsy Synergy

In classic presentations, either tissue can secure the diagnosis; in atypical or treatment-modified disease, using both increases yield.

- Muscle biopsy: look for perifascicular atrophy, perivascular/perimysial inflammation, capillary drop-out, and C5b-9 deposition consistent with a perifascicular microangiopathy. Add MxA immunohistochemistry, now widely recommended, because a positive perifascicular/sarcoplasmic MxA pattern is highly specific for DM and boosts diagnostic confidence when morphology is subtle [40,66].
- Skin biopsy: typical DM shows vacuolar interface dermatitis with dermal mucin; importantly, even spongiotic-appearing rashes may carry a type-I-IFN (MxA) signature, explaining why skin remains a reliable, minimally invasive window into systemic disease activity [67].

## 3. Discussion

While consistent trends were observed across studies, the overall evidence base remains heterogeneous. Many included cohorts were retrospective, single-center, and varied in diagnostic criteria and follow-up duration. Sample sizes were often small, particularly for emerging autoantibody subsets, limiting generalizability. Variability in imaging protocols, serologic panels, and treatment regimens further complicates pooled interpretation (Table 5) and underscores the need for larger, prospective multicenter studies.

### 3.1. Putting It Together

1. Suspect DM from skin findings ± proximal weakness → draw CK/aldolase and MSA panel.
2. If CK is normal or exam equivocal, obtain MRI (thighs/shoulder girdle) and NVC; and add EMG for localization.
3. Conduct a biopsy on the most MRI-active muscle; perform skin biopsy from a representative lesion. Include MxA IHC (muscle ± skin) when available.
4. Reconcile results with serology to assign the endotype and screen for ILD and malignancy per risk profile. This integrated approach of skin + muscle phenotype, aldolase when CK is normal, MRI-guided biopsy, and MxA-supported histology captures both classic and hypomyopathic disease while minimizing false negatives [64,65].

### 3.2. Treatment Strategies, Prognosis and Complications

First-line and steroid-sparing therapy: High-dose systemic glucocorticoids remain the initial anchor (e.g., prednisone ~0.5–1 mg/kg/day, with early taper), but current practice is to add a conventional immunosuppressant up front to limit steroid exposure and deepen response. Common choices include methotrexate, azathioprine, and mycophenolate mofetil, and these have the best overall balance of efficacy and familiarity across skin and muscle disease, with calcineurin inhibitors (tacrolimus or cyclosporine) favored when interstitial lung disease (ILD) is present. Recent dermatology-focused reviews support this stepwise backbone and emphasize aligning drug choice with the dominant phenotype (skin-predominant, myopathic, or ILD) and the myositis-specific autoantibody (MSA) profile [68].

IVIG and apheresis: Intravenous immunoglobulin (IVIG) is now an evidence-based standard therapy for active adult DM. In the phase-3 ProDERM randomized trial (*n* = 95), IVIG 2 g/kg every 4 weeks improved global myositis outcomes versus placebo and enabled steroid reduction; these data underpinned the 2021 USA FDA approval of Octagam 10% as the first approved DM treatment. Post hoc analyses also showed clinically meaningful skin improvement (CDASI) tracking with global response. Use 2 g/kg every 4 weeks during active disease, then space as tolerated [68,69]. For rapidly progressive or treatment-refractory IIM-ILD (especially anti-MDA5), therapeutic plasma exchange can be considered as an adjunct rescue. Recent studies and reviews suggest improved 1-year survival and radiographic/physiologic gains with plasma exchange when standard regimens are failing, though evidence remains low-moderate quality, and risks (infection, access complications) require careful selection [70,71].

Biologics and targeted agents:

Rituximab (anti-CD20): The RIM randomized placebo-phase trial did not meet its primary endpoint, yet 83% of refractory PM/DM/JDM patients achieved the myositis improvement definition overall; in practice, rituximab is widely used in refractory disease and select ILD phenotypes (ODDIS).

Abatacept (CTLA-4-Ig): A 2025 phase-3 trial of weekly subcutaneous abatacept plus standard care in refractory IIM showed no significant overall benefit at 24 weeks versus placebo, with a possible signal in non-DM subtypes; safety was acceptable. Consider selected, non-DM IIM after other options [3,69].

JAK inhibitors: Mounting observational data, including open-label adult DM cohorts and pediatric series, show meaningful improvements in skin (CDASI) and muscle activity with tofacitinib or baricitinib; emerging evidence in anti-MDA5 ILD suggests better 1-year transplant-free survival versus calcineurin-inhibitor-based regimens in high-risk cohorts. A 2024 meta-analysis supports effectiveness with acceptable safety, with vigilance for infections (e.g., CMV). Randomized data are expanding in JDM [72,73].

Prospects: Multiple late-breaking programs are testing interferon-axis and humoral-immunity targets in IIM/DM, including FcRn inhibitors (nipocalimab, efgartigimod), dual TYK2/JAK1 inhibition (brepocitinib), and TLR7/8/9 blockade (enpatoran). These reflect, and may personalize, treatment by serotype/phenotype in the near future [68].

### 3.3. Prognosis and Phenotype-Guided Care

Prognosis in dermatomyositis varies widely depending on systemic involvement and autoantibody status. In a recent population-based cohort, the standardized mortality ratio for myopathic dermatomyositis was approximately 3.1 (95% CI 1.1–6.8) compared with the general population [11]. Overall 5-year survival has been reported between 60% and 75%, with malignancy-associated and interstitial-lung-disease-associated dermatomyositis showing the poorest outcomes [7,11,50]. The most frequent causes of death include cancer-related complications, respiratory failure from ILD, and infections secondary to chronic immunosuppression. Patients with anti-MDA5 antibodies experience particularly aggressive, rapidly progressive ILD and shorter survival, whereas those with anti-TIF1-γ antibodies require vigilant malignancy surveillance. Early recognition and multidisciplinary management have improved survival over recent decades, yet relapse and chronic muscle weakness persist in up to one-third of patients. Outcomes hinge on extramuscular complications (ILD, malignancy) and the MSA profile. Anti-MDA5-associated ILD remains the main driver of early mortality; contemporary cohorts and guidance favor early multi-agent therapy and consideration of JAK inhibition in appropriate patients [74]. Cancer is the leading cause of death in adult IIM, and is concentrated within ~3 years around disease onset. IMACS 2023 provides a risk-stratified screening framework: basic screening for all adults at diagnosis; add an “enhanced” panel (e.g., CT, tumor markers) for moderate/high risk; and consider 18F-FDG PET-CT as a single screen in anti-TIF1-γ-positive DM onset >40 years with other high-risk features. Commonly detected tumors include lung, ovarian, colorectal, breast, lymphoma, and, in endemic regions, nasopharyngeal cancer [58]. AMA-positive myositis represents a rare but clinically distinctive form of inflammatory myopathy characterized by chronic disease course, preferential involvement of paraspinal and neck-extensor muscles, and frequent cardiac manifestations such as myocarditis, arrhythmia, and conduction block [14,20,21]. Histopathologic findings may include necrotizing or granulomatous features, while serology typically reveals the M2 AMA subtype. This entity is often associated with coexisting primary biliary cholangitis (PBC) and may respond variably to conventional immunosuppression, emphasizing the importance of cardiac monitoring and hepatology collaboration. Recognition of AMA-positive myositis broadens the IIM spectrum and highlights the overlap between myositis and systemic autoimmune diseases.

Recent data further emphasize that in AMA-positive myositis, cardiac involvement may be relatively refractory to standard immunosuppression: In a 2025 case-based review, persistent myocardial arrhythmias and conduction dysfunction were observed in a patient despite high-dose glucocorticoids and intravenous cyclophosphamide, and in the literature review, 41.7% of treated vs. 66.7% of untreated cases demonstrated deterioration of cardiac lesions [75]. This suggests that early cardiology involvement and tailored adjunctive strategies may be required.

#### Cutaneous vs. Myopathic Damage and Key Complications

Calcinosis cutis (especially in JDM, ≈20–40% lifetime prevalence) drives pain, ulceration, and disability. Evidence for therapy is low-level: case series support intralesional/topical/systemic sodium thiosulfate; observational DM/JDM reports suggest JAK inhibitors can soften calcinosis in some patients; surgical debulking remains pragmatic for focal burdens. Standardized outcome measures and trials are evolving [76,77]. Lipodystrophy (predominantly JDM) and chronic skin damage (poikiloderma, dyspigmentation) may persist despite myopathy control and are correlated with worse quality of life; CDASI damage/activity mapping is useful for tracking this domain alongside muscle strength and function [68]. ILD requires aggressive, early combination therapy; consider calcineurin inhibitor-based regimens, IVIG support, ± rituximab, or JAK inhibitor in selected phenotypes, and rescue plasma exchange if the disease is rapidly progressing. Multidisciplinary management (dermatology, rheumatology/neuromuscular, pulmonology) is central to improving survival and limiting organ damage [61,68,69].

The bottom line: Start steroids with an early steroid-sparing agent tailored to phenotype/MSA; add IVIG for active DM; escalate to rituximab/JAK inhibition (and abatacept selectively) when refractory; treat ILD aggressively, including apheresis when crashing, and screen for cancer using the 2023 IMACS risk-stratified protocol. This approach aligns with the best current evidence and the evolving precision-medicine landscape in DM treatment [58,69,78,79].

## 4. Materials and Methods

### 4.1. Protocol and Reporting

We conducted a narrative, evidence-focused review registered a priori as a concept in our lab notebook (not prospectively registered on PROSPERO). Methods and reporting follow PRISMA-2020 guidance for reviews without meta-analysis and the SANRA checklist for narrative reviews. No patient-level data were collected; IRB approval and consent were not required.

### 4.2. Research Question and Scope Objective

Our objective was to synthesize advances (2000–2025) in the cutaneous diagnosis, skeletal muscle involvement, immunopathogenesis, malignancy associations, and treatment of dermatomyositis (DM) and closely related inflammatory myopathies (juvenile DM, clinically amyopathic/hypomyopathic DM, anti-MDA5 DM, overlap phenotypes), with practical emphasis on skin-led recognition, MSA-guided endotyping, and phenotype-directed therapy (including ILD).

### 4.3. Eligibility Criteria Population/Condition

Eligible cases: Adults and children with dermatomyositis (classic, amyopathic/hypomyopathic, anti-MDA5), juvenile dermatomyositis, and relevant overlaps (e.g., anti-synthetase syndrome when DM-like skin findings are addressed), plus comparator IIMs when informing differential diagnosis (IMNM, scleromyositis). Concept/Outcomes: (i) diagnostic accuracy and characteristic features of cutaneous lesions, nailfold capillaroscopy, muscle MRI, EMG, skin/muscle histology (including MxA IHC); (ii) MSA-endotypes and associated risks (ILD, malignancy); (iii) treatment efficacy/effectiveness and safety (steroids, csDMARDs, IVIG, calcineurin inhibitors, rituximab, JAK/TYK2 inhibitors, apheresis); (iv) malignancy screening frameworks; and (v) pediatric-specific complications (calcinosis, lipodystrophy). Study designs (included): Randomized and non-randomized controlled trials, prospective/retrospective cohorts, case–control studies, large case series (*n* ≥ 10), diagnostic accuracy studies, translational/mechanistic studies, consensus guidelines, and high-quality systematic reviews/meta-analyses. Study designs (excluded): Single-patient case reports (except to highlight novel safety signals), narrative opinions without data, non-systematic overviews, non-human studies unless directly translational to human disease. Timeframe: 1 January 2000 to 15 August 2025. Language: English (and English-language abstracts with sufficient methodological detail).

### 4.4. Information Sources

We searched the available literature on MEDLINE (PubMed), Embase (Elsevier), Cochrane Library, Web of Science Core Collection, and Scopus, with publication dates from 2000 to 15 August 2025. To capture ongoing/late-breaking data, we screened ClinicalTrials.gov and recent abstracts from ACR/EULAR/AAD/ENMC (2021–2025). Reference lists of included articles and key guidelines (e.g., IMACS 2023) were hand-searched (“snowballing”). Gray literature was limited to major society statements and regulatory approvals relevant to DM therapy (e.g., IVIG).

### 4.5. Search Strategy

Search strategies combined controlled vocabulary and keywords. The PubMed strategy below was adapted for other databases (Emtree/Thesaurus terms and proximity operators adjusted). Filters: Humans; 1 January 2000–15 August 2025. PubMed (example): ((“Dermatomyositis” [Mesh] OR dermatomyositis [tiab] OR “clinically amyopathic dermatomyositis” [tiab] OR CADM [tiab] OR “juvenile dermatomyositis” [tiab] OR JDM [tiab]) AND (skin [tiab] OR cutaneous [tiab] OR heliotrope [tiab] OR Gottron* [tiab] OR poikiloderma [tiab] OR nailfold [tiab] OR capillaroscop* [tiab] OR “myxovirus resistance protein A” [tiab] OR MxA [tiab] OR biopsy [tiab] OR histolog* [tiab] OR “magnetic resonance imaging” [tiab] OR MRI [tiab] OR electromyograph* [tiab] OR EMG [tiab])) OR ((“myositis” [Mesh] OR “idiopathic inflammatory myopathy” [tiab] OR IIM [tiab]) AND (antisynthetase [tiab] OR “immune-mediated necrotizing myopathy” [tiab] OR IMNM [tiab]) AND (differential [tiab] OR MRI [tiab] OR biopsy [tiab])) OR ((“autoantibodies” [Mesh] OR autoantibody* [tiab] OR “myositis-specific autoantibod*” [tiab] OR MSA [tiab]) AND (MDA5 [tiab] OR “melanoma differentiation-associated protein 5” [tiab] OR “TIF1-γ” [tiab] OR TIF1G [tiab] OR NXP2 [tiab] OR Mi-2 [tiab] OR SAE [tiab] OR “Jo-1” [tiab])) OR ((“interstitial lung disease” [Mesh] OR ILD [tiab] OR “rapidly progressive” [tiab]) AND (MDA5 [tiab] OR dermatomyositis [tiab])) OR ((“immunoglobulins, intravenous” [Mesh] OR IVIG [tiab] OR rituximab [tiab] OR “calcineurin inhibitor*” [tiab] OR tacrolimus [tiab] OR cyclosporine [tiab] OR tofacitinib [tiab] OR baricitinib [tiab] OR JAK [tiab] OR TYK2 [tiab] OR abatacept [tiab] OR “plasma exchange” [tiab] OR plasmapheresis [tiab]) AND (dermatomyositis [tiab])) OR ((“neoplasms” [Mesh] OR malignanc* [tiab] OR cancer [tiab]) AND dermatomyositis [tiab] AND (screen* [tiab] OR IMACS [tiab])).

### 4.6. Study Selection

Two reviewers (AP, DR) independently screened titles/abstracts, then full texts, in Rayyan with masking enabled. Disagreements were resolved by consensus or third-reviewer adjudication (DL). We documented exclusion reasons during a full-text review (e.g., wrong population, inadequate data, duplicate cohort/overlap). When multiple reports described the same cohort, we used the most complete/updated analysis and cross-checked for overlapping participants.

### 4.7. Data Extraction

A piloted extraction form (AP, DR) captured the following data. Bibliographic details: country; enrollment years; design and sample size; phenotype (classic DM, ADM/HDM, anti-MDA5 DM, JDM, overlap); and age/sex. Diagnostics: cutaneous signs; nailfold features; skin/muscle histology (MxA/C5b-9); MRI sequences/findings (T2/STIR edema; T1 fatty replacement, distribution patterns); EMG features; and assay platforms for MSAs. Treatments: regimen; dose; timing/sequence (e.g., IVIG 2 g/kg q4wk); co-therapies; and rescue measures (apheresis). Outcomes: muscle (MMT-8, MYOACT, CK/aldolase); skin (CDASI activity/damage); lung (FVC, DLCO, HRCT pattern, transplant-free survival); global/IMACS DOI; and adverse events. Malignancy screening: risk tiering; modality; detection yield/timing; and pediatric-specific outcomes (calcinosis, lipodystrophy). Extraction was performed in duplicate (AP primary; DR secondary) with verification by DL for key efficacy/diagnostic tables.

### 4.8. Quality Assessment (Risk of Bias)

Design-appropriate tools were applied. Randomized trials: Cochrane RoB 2. Non-randomized interventional/observational cohorts: ROBINS-I; Newcastle–Ottawa Scale (NOS) for cohort/case–control. Diagnostic accuracy studies: QUADAS-2 (pre-specified index tests: MxA IHC, MRI, nailfold capillaroscopy, DIF/histology where applicable). Systematic reviews/meta-analyses: AMSTAR 2. Translational/mechanistic studies: adapted NIH Study Quality Assessment Tools (clarity of objectives, selection bias, assay validity/reproducibility, confounding). Risk-of-bias judgments informed the weight of each study and were received in synthesis; we did not exclude studies solely based on quality unless fatal flaws were identified (e.g., misclassification).

### 4.9. Evidence Synthesis and Handling of Heterogeneity

Given expected methodological and clinical heterogeneity (phenotypes, endpoints, assays), we performed a structured narrative synthesis rather than meta-analysis. Results are organized by domain (classification/endotypes; cutaneous diagnosis; skeletal muscle imaging/biopsy; immunopathogenesis; ILD and malignancy risk; and treatment classes). When trials or comparable cohorts reported common outcomes (e.g., CDASI change with JAK inhibition; IMACS DOI with IVIG), we present ranges and directionality with attention to risk of bias and precision. Subgroup emphasis was placed on phenotype (classic vs. ADM/HDM vs. JDM; anti-MDA5 vs. non-MDA5); organ domain (skin, muscle, ILD); antibody-defined risk (TIF1-γ/NXP2 malignancy risk; MDA5-ILD). Where quantitative pooling was infeasible, we prioritized consistency of effects across high-quality sources and triangulated with mechanistic plausibility.

### 4.10. Certainty/Strength of Evidence

For key clinical questions (e.g., IVIG effectiveness in adult DM; JAK inhibitors in DM skin activity and anti-MDA5 ILD survival; diagnostic utility of MxA IHC), we applied a pragmatic GRADE assessment (high/moderate/low/very low) based on study design, risk of bias, inconsistency, indirectness, imprecision, and publication bias. GRADE ratings are summarized alongside recommendations in the Discussion.

### 4.11. Publication Bias and Selective Reporting

We qualitatively considered asymmetry (e.g., positive small studies in emerging therapies) and preferential reporting in conference abstracts; peer-reviewed and full-text data were weighted more heavily. Conflicts of interest: Industry sponsorship and investigator conflicts were recorded when stated and considered during the weighting process.

### 4.12. Role of the Funders

No external funding influenced the design, study selection, data extraction, appraisal, synthesis, or decision to publish.

## 5. Conclusions

Future Directions and Conclusion: Future work in dermatomyositis should center on precision medicine driven by myositis-specific autoantibodies: anti-MDA5 guiding early ILD-focused regimens (often including JAK inhibition), anti-TIF1-γ or anti-NXP2 prompting intensified, time-bound cancer screening, and anti-Mi-2 favoring skin-predominant steroid-sparing strategies. Biomarker development should prioritize interferon-linked serum markers (for example, CXCL10, galectin-9), lesional MxA immunohistochemistry, and nailfold capillaroscopy, integrated with MRI activity scores to detect flare earlier than creatine kinase and to individualize tapering. Skin should be leveraged as a minimally invasive window into systemic disease using standardized CDASI activity/damage scoring and serial imaging, reducing reliance on repeat muscle biopsy. Therapeutic pipelines will likely expand through JAK/TYK2 inhibitors for IFN-high endotypes, FcRn inhibitors, and B-cell or co-stimulation blockade for refractory disease, and trials stratified by antibody and ILD status. Parallel advances in radiomics/AI for MRI patterning, remote photo/NVC monitoring, and outreach models for rural/underserved patients can shorten time to diagnosis and escalation. In conclusion, dermatomyositis is an interferon-driven microangiopathy with tightly coupled skin–muscle inflammation and autoantibody-defined endotypes. Optimal outcomes are determined by early recognition of cutaneous clues, rapid integration of serology with MRI/EMG and MxA-supported histology, prompt initiation of steroid-sparing therapy (with IVIG for active disease and targeted agents when indicated), and risk-stratified screening for interstitial lung disease and malignancy. Consistent, interdisciplinary care among dermatology, rheumatology/neuromuscular medicine, pulmonology, and oncology remains the cornerstone of durable control while minimizing chronic skin damage and muscle disability. Despite recent advances, standardized biomarkers for disease activity and progression remain lacking, limiting reproducibility across studies and clinical settings [6,14]. Continued efforts are needed to incorporate comprehensive autoantibody profiling into diagnostic algorithms, including emerging and rarer markers such as anti-mitochondrial antibodies (AMAs) and anti-PM/Scl [20,21]. Integrating these profiles into prospective cohorts may help refine phenotypic classification, enable earlier identification of atypical cases, and support precision-medicine approaches once validated in larger studies [6,15].

## Figures and Tables

**Table 2:** Classic cutaneous lesions in dermatomyositis: Key features, photography tips, optimal biopsy targets, histopathology, and common pitfalls: This table summarizes hallmark dermatomyositis skin findings with practical documentation and biopsy guidance. “Best photograph angle” prioritizes clinic-ready images that capture diagnostic morphology and distribution. “Best biopsy target” indicates the preferred site and depth for routine histopathology; sample the freshest, non-excoriated lesion when possible. Histopathologic descriptors refer to routine hematoxylin–eosin findings; dermal mucin may be highlighted with special stains when needed. Periungual capillary changes are best evaluated noninvasively via dermoscopy or videocapillaroscopy procedures. Use the “Common pitfalls/differentials” column to avoid misclassification of photo-distributed or frictional dermatoses. Abbreviations: metacarpophalangeal (MCP); interphalangeal (IP); subacute cutaneous lupus erythematosus (SCLE); cutaneous T-cell lymphoma (CTCL). Notes: (a) Avoid ulcerated or heavily lichenified areas when selecting a biopsy site. (b) Periorbital biopsy for the heliotrope rash is rarely necessary; if tissue is required, consider sampling an involved photo-distributed plaque at a safer site.

Lesion	Typical Anatomic Site(s)	Key Visual Features	Best Photograph Angle	Best Biopsy Target (Site/Depth)	Expected Histopathology	Common Pitfalls/Differentials
Gottron’s papules	Dorsal MCP/IP joints; elbows; knees	Violaceous-to-erythematous, flat-topped papules/plaques over extensor prominences; may have scale and erosions.	Dorsal hand close-up with raking light; include multiple joints for context.	Fresh, non-ulcerated papule on dorsal MCP; 3–4 mm punch to mid-dermis (avoid thick callus).	Vacuolar interface dermatitis, epidermal atrophy, basement-membrane thickening; superficial perivascular lymphocytes; increased dermal mucin.	Psoriasis over joints, lichen planus, chronic eczema/irritant dermatitis, knuckle pads.
Gottron sign (macular)	Extensor surfaces over elbows, knees; dorsal hands	Macular violaceous erythema without discrete papules; accentuated over bony prominences.	Orthogonal mid-range plus close-up of one joint.	Erythematous plaque on elbow/knee; 3–4 mm punch including epidermis and superficial dermis.	Interface change with basal vacuolization; superficial perivascular lymphocytes; dermal mucin.	Subacute cutaneous lupus erythematosus (SCLE), photodermatitis, irritant dermatitis from pressure/friction.
Heliotrope rash	Periorbital skin and upper eyelids	Violaceous periorbital erythema often with eyelid edema; may have fine scale.	Frontal portrait at eye level; neutral expression; remove makeup/eyewear.	Biopsy rarely required; if necessary, small punch from lateral upper eyelid by experienced surgeon; alternatively sample an involved photo-distributed plaque elsewhere.	Subtle vacuolar interface dermatitis; superficial perivascular lymphocytic infiltrate; dermal mucin.	Atopic/contact eyelid dermatitis, seborrheic dermatitis, blepharitis, lupus malar/eyelid involvement.
Poikiloderma (photo-distributed)	Anterior chest (V-sign), posterior neck/shoulders (shawl), lateral thighs (holster)	Reticulated hypo-/hyperpigmentation with telangiectasias and epidermal atrophy; often pruritic.	Anatomic overview plus close-up with macro lens to capture telangiectasias/atrophy.	Representative poikilodermatous plaque on chest or shoulder; 4 mm punch to mid-dermis.	Interface dermatitis with epidermal atrophy, pigment incontinence, telangiectasias; dermal mucin.	Poikiloderma of Civatte, chronic actinic damage, SCLE, mycosis fungoides (patch-stage).
Periungual erythema and nailfold capillary changes	Proximal nailfolds of fingers (±toes)	Dilated/tortuous capillary loops, drop-out, periungual erythema, ragged cuticles; often tender.	Dermoscopic or macro images at 20–30° oblique; include ruler for scale.	Biopsy not typically indicated—prefer noninvasive nailfold dermoscopy/videocapillaroscopy.	If biopsied: interface dermatitis and superficial perivascular lymphocytes; vascular ectasia.	Scleroderma spectrum capillaropathy, Raynaud-related changes, trauma/picking, psoriasis paronychia.

**Table 3:** Photo-accentuated distribution patterns in dermatomyositis (shawl, V, and holster signs): Diagnostic use, triggers, biopsy decision points, and key differentials. Shawl, V, and holster signs are characteristic distribution patterns that support the diagnosis of dermatomyositis when interpreted with pathognomonic lesions (for example, Gottron’s papules or heliotrope rash) and serology. “Common triggers” highlight factors that intensify photo-accentuated disease. “When to biopsy versus photograph only” offers pragmatic guidance: photograph typical lesions for longitudinal comparison; biopsy if plaques are atypical, indurated, ulcerated, or if lymphoma, psoriasis, or lupus is in the differential. The holster sign is often less sun-dependent than shawl or V patterns and warrants biopsy if persistent or asymmetric. Abbreviations: subacute cutaneous lupus erythematosus (SCLE); cutaneous T-cell lymphoma (CTCL); mycosis fungoides (MF). Notes: (a) Capture both an anatomic overview and a dermoscopic or macro close-up to document telangiectasias and atrophy. (b) Consider concomitant nailfold imaging when systemic involvement is suspected.

Pattern	Distribution	Common Triggers	Diagnostic Implications	When to Biopsy vs. Photograph Only	Key Differentials
Shawl sign	Posterior neck, shoulders, upper back (photo-accentuated).	Ultraviolet exposure, heat	Highly characteristic for dermatomyositis; supports diagnosis when combined with Gottron/heliotrope.	Photograph for documentation; biopsy if lesion is atypical, indurated, or to exclude SCLE/CTCL.	SCLE, polymorphous light eruption, actinic dermatitis, poikiloderma of Civatte.
V-sign	Anterior neck and upper chest in a V-shaped photo-distribution.	Ultraviolet exposure	Characteristic but not pathognomonic; consider with other DM stigmata.	Usually photograph; biopsy a representative plaque if features are atypical.	Phototoxic drug eruption, SCLE, tinea versicolor (dyspigmentation), seborrheic dermatitis.
Holster sign	Lateral thighs/hips (often less sun-exposed).	May be frictional or heat-exacerbated; not strictly photo-accentuated	Helpful corroborating sign for DM, especially with other lesions.	Photograph; biopsy if indurated, atypical, or to rule out MF/eczema/psoriasis.	Nummular eczema, psoriasis, tinea corporis, early mycosis fungoides.

**Table 5:** Integrated diagnostic approach for dermatomyositis: Modalities include examination, serum enzymes, myositis autoantibodies, skeletal muscle MRI, electromyography, skin biopsy, muscle biopsy, and nailfold videocapillaroscopy. For each, the table summarizes primary role, typical positive findings, strengths, limitations/pitfalls, and actions when results are normal or discordant. Abbreviations: anti–MDA5 = melanoma differentiation-associated protein 5; anti–TIF1-γ = transcription intermediary factor 1-gamma; anti–NXP2 = nuclear matrix protein 2; anti–SAE = small ubiquitin-like modifier activating enzyme; MxA = myxovirus-resistance protein A.

Modality	Primary Role	Typical Positive Findings	Strengths	Limitations and Pitfalls	Action When Results Are Normal or Discordant
Dermatologic and neurologic examination	Identify pathognomonic skin lesions and symmetric proximal weakness; screen for dysphagia and respiratory involvement.	Gottron’s papules or Gottron sign; heliotrope rash; photo-accentuated poikiloderma; periungual capillary changes; proximal shoulder and hip girdle weakness.	Immediate, noninvasive, high specificity when classic skin lesions are present.	Cutaneous signs can be subtle or treatment-modified; early hypomyopathic disease may lack objective weakness.	Proceed to laboratory testing, magnetic resonance imaging of skeletal muscle, and consider skin biopsy.
Serum enzymes	Detect myocyte injury and estimate activity.	Elevated creatine kinase; elevated aldolase; possible elevation of lactate dehydrogenase and transaminases.	Widely available; dynamic relationship with disease activity.	Creatine kinase can be normal in hypomyopathic and anti–MDA5 phenotypes; isolated aldolase elevation may be the only clue.	If enzymes are normal but suspicion remains, obtain muscle magnetic resonance imaging and electromyography; consider tissue biopsy.
Myositis-specific and myositis-associated autoantibodies	Define endotype and guide risk (for example, malignancy or interstitial lung disease).	Anti–Mi-2, anti–TIF1-γ, anti–NXP2, anti–MDA5, anti–SAE, or anti-synthetase antibodies.	Improves diagnostic confidence; predicts organ involvement; informs cancer screening.	Commercial panels vary; seronegativity does not exclude disease.	Interpret with clinical, imaging, and histology; do not delay urgent treatment while awaiting extended panels.
Magnetic resonance imaging of skeletal muscle	Detect active inflammation and guide biopsy site selection.	Bilateral, symmetric T2/STIR hyperintensity in proximal muscles and fascia; T1 hyperintensity and atrophy in chronic damage; anterior thigh predominance pattern typical for dermatomyositis.	Sensitive for subclinical disease; distinguishes activity from chronic fatty replacement; supports subtype distinction versus necrotizing myopathy.	Cost and availability; edema patterns can overlap with other myopathies or denervation.	If magnetic resonance imaging is inactive but suspicion persists, re-examine the skin, repeat enzymes, consider whole-body or alternative-site imaging, and correlate with electromyography.
Electromyography	Demonstrate active myopathy and help localize a biopsy.	Short-duration, low-amplitude motor unit potentials with early recruitment; fibrillation potentials and positive sharp waves.	Physiologic evidence of active membrane irritability; complements imaging.	Sensitivity is imperfect; may be normal early or in hypomyopathic disease; uncomfortable for patients.	Use alongside magnetic resonance imaging for localization rather than to exclude disease; proceed to biopsy if suspicion remains high.
Skin biopsy	Minimally invasive tissue confirmation and biomarker window into systemic activity.	Vacuolar interface dermatitis with epidermal atrophy and increased dermal mucin; capillary ectasia; positive myxovirus-resistance protein A (MxA) immunostaining when available.	High yield when classic lesions are sampled; lower risk than muscle biopsy.	Histology can overlap with cutaneous lupus; site selection is critical; eyelid biopsy is rarely indicated.	If nondiagnostic, obtain muscle magnetic resonance imaging and muscle biopsy; repeat skin biopsy from a more active site if needed.
Muscle biopsy (with immunohistochemistry)	Gold-standard anatomic diagnosis when inflammatory myopathy is suspected.	Perifascicular fiber atrophy; perivascular and perimysial inflammation; capillary drop-out with C5b-9 deposition; positive myxovirus-resistance protein A staining supports a type I interferon signature.	Defines the perifascicular microangiopathy of dermatomyositis and separates it from other inflammatory myopathies.	Yield depends on site; prior treatment may blunt inflammation; invasive procedure.	Use magnetic resonance imaging and electromyography to guide site; add or repeat myxovirus-resistance protein A and complement staining; repeat biopsy if the clinical course is strongly suggestive.
Nailfold videocapillaroscopy	Noninvasive assessment of microangiopathy and disease activity.	Dilated and tortuous loops, capillary drop-out, periungual erythema and hemorrhages.	Quick, repeatable, helpful for longitudinal monitoring.	Operator dependent; not specific and overlaps with scleroderma spectrum.	Use as an adjunct; do not use in isolation to exclude disease.

## Data Availability

No new data were created or analyzed in this study.

## Abbreviations

18F-FDG	Fluorodeoxyglucose (for PET imaging)
ACR	American College of Rheumatology
ADM	Amyopathic dermatomyositis
AEs	Adverse events
AMA	Against medical advice (if used); otherwise omit
ASyS	Anti-synthetase syndrome
C5b-9 (MAC)	Membrane–attack complex (complement)
CADM	Clinically amyopathic dermatomyositis
CDASI	Cutaneous Dermatomyositis Disease Area and Severity Index
CK	Creatine kinase
CMV	Cytomegalovirus
CRP	C-reactive protein
CT	Computed tomography
CTCL	Cutaneous T-cell lymphoma
CXCL10	C-X-C motif chemokine ligand 10
DLCO	Diffusing capacity of the lungs for carbon monoxide
DM	Dermatomyositis
DMARD	Disease-modifying antirheumatic drug
csDMARD	Conventional synthetic disease-modifying antirheumatic drug
DIF	Direct immunofluorescence
EMG	Electromyography
ENMC	European Neuromuscular Centre
ESR	Erythrocyte sedimentation rate
EULAR	European Alliance of Associations for Rheumatology
FDA	US Food and Drug Administration
FVC	Forced vital capacity
GRADE	Grading of Recommendations, Assessment, Development and Evaluations
HDM	Hypomyopathic dermatomyositis
HRCT	High-resolution computed tomography
IBM	Inclusion body myositis
IHC	Immunohistochemistry
IIM	Idiopathic inflammatory myopathy
ILD	Interstitial lung disease
IMACS	International Myositis Assessment and Clinical Studies Group
IMNM	Immune-mediated necrotizing myopathy
IRB	Institutional Review Board
ISG	Interferon-stimulated gene
IVIG	Intravenous immunoglobulin
JAK	Janus kinase
JDM	Juvenile dermatomyositis
JIIM	Juvenile idiopathic inflammatory myopathy
LDH	Lactate dehydrogenase
MF	Mycosis fungoides
Mi-2 (anti–Mi-2)	Mi-2 autoantibody
MRI	Magnetic resonance imaging
MSA	Myositis-specific autoantibody
MAA	Myositis-associated autoantibody
MMT-8	Manual Muscle Testing-8
MxA	Myxovirus-resistance protein A
MYOACT	Myositis Disease Activity Assessment Tool
NPV/PPV	Negative/Positive predictive value (if used)
NSIP	Nonspecific interstitial pneumonia
NVC	Nailfold videocapillaroscopy
NXP2 (anti–NXP2)	Nuclear matrix protein 2 autoantibody
PET/CT	Positron emission tomography/computed tomography
PM	Polymyositis
PM/Scl (anti–PM/Scl)	Polymyositis/scleroderma autoantibody
PRISMA	Preferred Reporting Items for Systematic Reviews and Meta-Analyses
ProDERM	Phase 3 IVIG trial in dermatomyositis
qPCR (if used)	Quantitative polymerase chain reaction
RIM	Rituximab in Myositis trial
RP-ILD	Rapidly progressive interstitial lung disease
SAE (anti–SAE)	Small ubiquitin-like modifier activating enzyme autoantibody
SCLE	Subacute cutaneous lupus erythematosus
SSc	Systemic sclerosis
SPEP	Serum protein electrophoresis
SRP (anti–SRP)	Signal recognition particle autoantibody
STIR	Short tau inversion recovery (MRI sequence)
T1/T2	T1- and T2-weighted MRI sequences
TIF1-γ (anti–TIF1-γ)	Transcription intermediary factor 1-gamma autoantibody
TYK2	Tyrosine kinase 2
WB-MRI	Whole-body magnetic resonance imaging
